# Prognostic significance of negative lymph node count in microsatellite instability-high colorectal cancer

**DOI:** 10.1186/s12957-024-03469-4

**Published:** 2024-07-19

**Authors:** Xuan Dai, Zhujiang Dai, Jihong Fu, Zhonglin Liang, Peng Du, Tingyu Wu

**Affiliations:** Department of Colorectal and Anal Surgery, Xinhua Hospital, Shanghai Jiao Tong University School of Medicine, Shanghai, China

**Keywords:** Negative lymph node, Microsatellite instability-high, Colorectal cancer, Lymph-Node Ratio, Lymph node metastasis, Prognostic factor

## Abstract

**Background:**

Microsatellite instability-high (MSI-H) tumors, with elevated tumor mutational burden and expression of neoantigens, represent a distinct immune-activated subpopulation in colorectal cancer (CRC), characterized by strong lymph node reaction, locally advanced tumor and higher total lymph nodes harvested (TLN), but less metastatic lymph nodes and fewer incidence of III-IV stage. Host immune response to tumor and lymph nodes may be an important prognostic factor. However, N stage and LNR (Lymph-Node Ratio) have limitations in predicting the prognosis of MSI-H patients. Negative lymph node count (NLC) provided a more precise representation of immune activation status and extent of tumor metastasis. The study aims to detect prognostic significance of NLC in MSI-H CRC patients, and compare it with N stage, TLN and LNR.

**Methods:**

Retrospective data of 190 consecutive MSI-H CRC patients who received curative resection were collected. Survival analyses were performed using the Kaplan–Meier method. Clinicopathological variables including NLC, N stage, TLN and LNR were studied in univariate and multivariate COX regression analyses. ROC (receiver operating characteristic curve) and concordance index were employed to compare the differences in predictive efficacy between NLC, N stage, TLN and LNR.

**Results:**

Patients with increased NLC experienced a significantly improved 5-years DFS and OS in Kaplan–Meier analysis, univariate analysis, and multivariate analysis, independent of potential confounders examined. Increased NLC corresponded to elevated 5-years DFS rate and 5-years OS rate. AUC (area under curve) and concordance index of NLC in DFS and OS predicting were both significantly higher than N stage, TLN and LNR.

**Conclusions:**

Negative lymph node is an important independent prognostic factor for MSI-H patients. Reduced NLC is associated with tumor recurrence and poor survival, which is a stronger prognostic factor than N stage, TLN and LNR.

## Introduction

Microsatellite instability-high (MSI-H) tumors, which caused by inactivation of the mismatch repair systems due to deficiency of mismatch repair genes (dMMR), represent a distinct pathological subtype in colorectal cancer (CRC) [1]. Whereas tumors could be considered as microsatellite stable (MSS) if no mutation of mismatch repair genes could be detected. MSI-H/dMMR CRC have a distinct phenotype characterized by an increased mutational burden, mucinous histology, poor differentiation and right colon segment location [2]. More importantly, MSI-H/dMMR CRC has been referred to as the “hot tumor” due to extensive immune cell infiltration, strong tumor immune response and favorable response to immunotherapy [3]. A stronger host immune response and increased lymphocytic infiltration within tumor indicate improved outcomes for immunotherapy and a more favorable prognosis [4–7].

Lymph node metastasis (N stage) is the single most important prognostic factor in CRC [8]. However, N stage has some limitations in predicting the prognosis of MSI-H/dMMR patients, whose tumors are likely to proliferate and progress locally, but are less likely to develop lymph node or distant metastasis with strong lymph node reaction [9–12]. For instance, many MSI-H/dMMR patients with locally advanced tumors and poor prognosis still do not develop lymph node metastases, thus the predictive value of traditional N stage is limited.

In MSI-H/dMMR patients with “hot tumor” reaction, higher lymph node harvest in CRC resection specimens indicates stronger immune response and more favorable prognosis [9]. To more accurately predict the prognosis of MSI-H/dMMR patients, the total number of lymph nodes need to be included, in addition to metastatic lymph nodes, for a comprehensive assessment.

Previous studies have shown that an increased ratio between metastatic lymph nodes and total lymph nodes harvested in the specimen (Lymph-Node Ratio, LNR) has been associated with a worse prognosis, displaying this ratio as the stronger prognostic factor of CRC related survival [13–17]. However, the LNR has no predictive significance for stage I—II patients with negative metastatic lymph nodes, since the LNR value remains at 0. The predictive value of LNR is limited in the MSI patients due to the higher prevalence of stage II cases.

The negative lymph node count (NLC), which assessed both the number of total lymph nodes and the metastatic lymph nodes, may serve as a prognostic factor for patients. Previous study has shown that the number of negative nodes is a prognostic factor for patients with stage IIIB and IIIC colon cancer, however, there was no association between the NLC and DFS for patients with stage IIIA disease [18]. The mechanisms underlying the relationship between the lymph node count and survival remain uncertain. The number of lymph nodes may be an indicator of host immune response to tumor cells [18, 19]. The highly activated anti-tumor immune response in MSI-H tumors may suggest a potentially superior prognostic predictive value for NLC. In MSI-H gastric cancer patients, higher NLC showed Improved DFS [20]. However, the prognostic value of NLC in MSI-H colorectal cancer has not been reported in relevant studies.

The purpose of this paper was to investigate prognostic significance of negative lymph node count in MSI-H/dMMR CRC patients, and compare it with N stage, total lymph node and LNR.

## Materials and Methods

### Study design, setting and population

**Fig.1:**
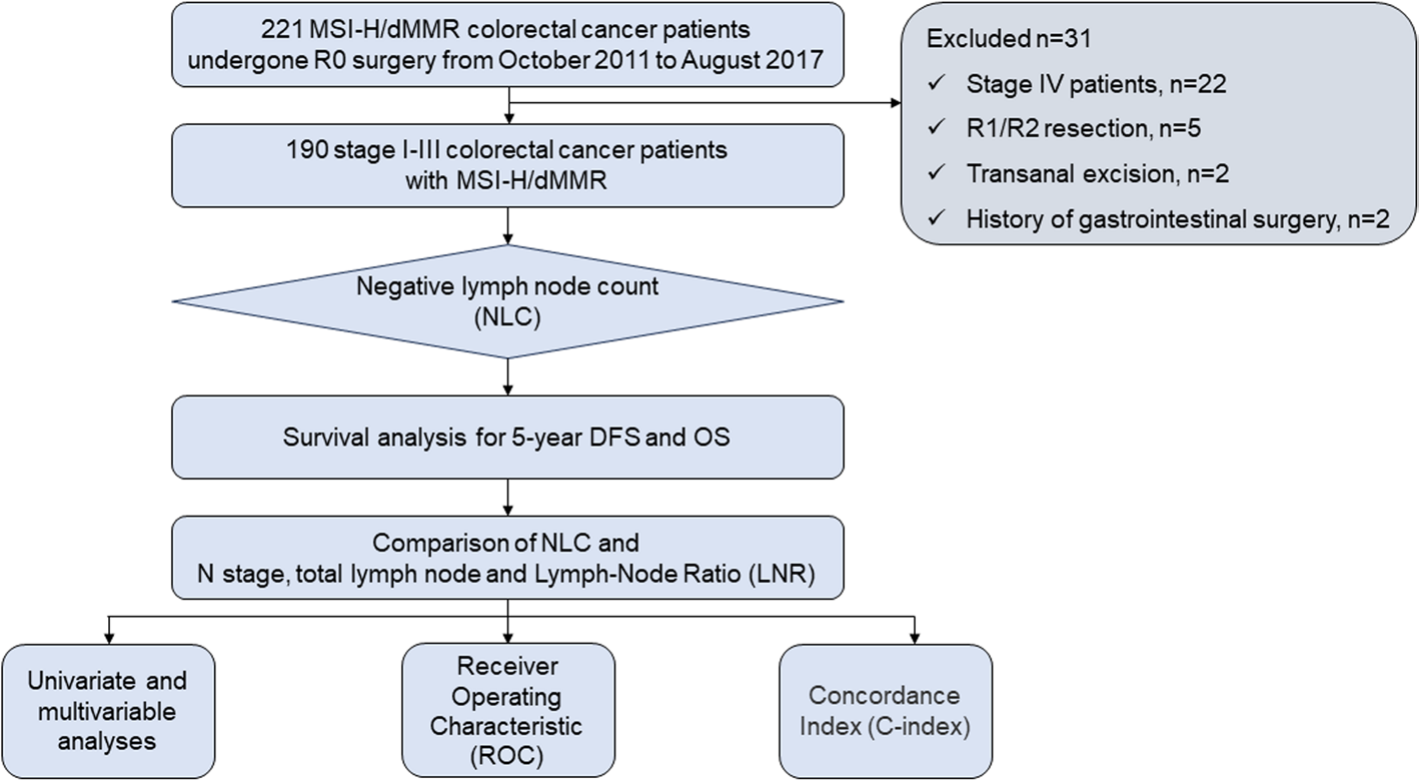
Diagram representing the selection and analysis of the study population

Between October 2011 and August 2017, retrospective data of 221 consecutive colorectal cancer patients who received curative resection and MSI-H/dMMR status were collected from the database of the department of colorectal and anal surgery, xinhua hospital, shanghai jiao tong university school of medicine (Figs. 1). The exclusion criteria were as follows: (1) Stage IV patients (*n* = 22); (2) R1/R2 resection (*n* = 5); (3) Transanal excision (*n* = 2); (4) History of gastrointestinal surgery (*n* = 2). After inclusion and exclusion, a total of 190 MSI-H/dMMR CRC patients with R0 resection were included in this study for retrospective analysis. Reviewed records included: patient’s baseline clinical demographics, tumor location, type of surgical procedures, pathological data and prognosis of survival and recurrence. The study was conducted in accordance with the Declaration of Helsinki, and approved by the ethics committee of xinhua hospital affiliated to shanghai jiaotong university school of medicine (approval No. XHEC-D-2023–176). Signed consents for the treatment and evaluation of data were obtained from all patients.

### Follow-Up

Follow-up of the patients has been updated yearly by telephone interviews, imaging, pathology, or CEA yearly by the surgical team, with the following end-points: overall survival (any cause of death or the last follow-up) and disease free survival (first recurrence after surgical treatment or the last follow-up). The follow-up evaluation of this study ended on August, 2022. The mean follow-up time was 74 months (95% CI, 36—95.8 months), and this study included 60 months of survival data for analysis due to more complete 5-year follow-up data.

### Microsatellite analysis

Immunohistochemistry (IHC) staining of four kinds of mis-match repair (MMR) protein (MLH1, MSH2, MSH6, PMS2) were used to determine MMR status. Negative expression of one or more of these proteins was determined to be deficiency of MMR (dMMR). DNA from paraffin-embedded tissue were also extracted, and MSI status was determined using microsatellite markers (NR21, NR27, BAT25, BAT26, MONO27, NR24). MSI-high was defined as the presence of instability in ≥ 30% of the markers (or ≥ 2 microsatellite loci), and MSI-low/ MSS as no or < 30% (or ≤ 1 microsatellite loci) unstable markers.

### Evaluation of Lymph Nodes

The pathological data reported the number of total lymph node harvest (TLN) and the number of metastatic lymph nodes in each resection. N stage (N0, N1 and N2) is defined by the TNM stage based on American Joint Committee on Cancer classification version 8. The LNR was defined as the number of metastatic lymph nodes divided by the total number of all examined nodes in the specimens. The patients were stratified into 4 subgroups and each group comprised the following LNR: first quartile, LNR1: < 0.12; second quartile, LNR2: 0.12 to 0.18; third quartile, LNR3: 0.18 to 0.40, and fourth quartile, LNR4: > 0.40 [14]. Negative lymph node count (NLC) was defined as the number of total lymph nodes subtracted by the number of metastatic lymph nodes. NLC was stratified on quartiles into 4 categories: first quartile, NLC1: 0–9; second quartile, NLC2: 10—13; third quartile, NLC3: 14—17; and fourth quartile, NLC4: ≥ 18. Total lymph node harvest (TLN) is stratified into 2 categories: TLN1: < 12; TLN2: ≥ 12 [14, 16].

### Statistical analysis

ANOVA test and Kruskal–Wallis test were used for quantitative variables with normal and nonnormal distribution. Pearson's χ2 test and Fisher’s exact test were used for nominal variables. 5-years DFS and OS were analyzed by the Kaplan–Meier method and compared using the log-rank test. Multivariate COX regression model was used to evaluate the risk factors of DFS and OS, adjusting for covariates determined a priori to be clinically relevant. These covariates included age, gender, tumor location, pathology, differentiation grade, tumor size, T stage, N stage, LNR, total lymph node and negative lymph node count. The receiver operating characteristic curve (ROC) is a curve that combines sensitivity and specificity and can be used to evaluate the predictive validity of an indicator. Area under curve (AUC) was defined as the geometric area to the lower right of the ROC curve and is used to quantify the predictive efficacy of the indicator. The C-index (concordance index) is also employed to assess the predictive capability of prognostic indicators, primarily utilized in the survival analysis to measure the discrimination between COX model predicted values and actual outcomes. The C-index represents the proportion of patients whose predicted outcomes match the actual outcomes among the entire patient population. The C-index provides a concise summary of three distinct dimensions of survival prediction (risk, event occurrence and time). The C-index change enables comparison of prediction accuracy between two prediction models. The AUC value and C-index of 0.5–0.7 represented low predictive efficacy, 0.7–0.9 indicated moderate predictive efficacy, and > 0.9 represented high predictive efficacy. In this study, ROC and C-index were employed to compare the differences in predictive efficacy between NLC, N stage, LNR and total lymph node. Statistical analysis was performed using SPSS 27 software and R programming language 4.3.1. Statistical significance was defined at P values < 0.05.

## Result

### Baseline characteristics and number of negative lymph nodes in CRC patients

A total of 190 MSI-H/dMMR CRC patients with R0 curative resection were included in this study (114 males, 76 female) (Table 1). 8 (4.2%) patients had stage I cancers, 94 (49.5%) patients had stage II cancers, and 88 (46.3%) patients had stage III cancers. Mean age at the time of surgery was of 63.7 years (SD, 12.7). Negative lymph node count (NLC) was stratified on quartiles into 4 categories (NLC1: 0–9; NLC2: 10—13; NLC3: 14—17; NLC4: ≥ 18). Table 1 shows the baseline demographics and clinicopathologic characteristics of MSI-H/dMMR CRC patients, comparing the differences between different NLC subgroups. Right side tumor location and larger tumor size showed a significant association with the higher negative lymph node count (*p* = 0.014 and 0.018, respectively). Moreover, we noted a trend of younger patients in the NLC4 group comparing NLC1 group (*p* = 0.017). There were no significant differences in gender, pathology, differentiation grade, T stage and surgical procedures among the four groups.

**Table 1:** Baseline demographics and clinicopathologic characteristics of the MSI-H/dMMR CRC patients

**Variable**	**All cases** (***n*** = 190)	**Negative lymph node count (%)**				
		**NLC1: 0–9** (***n*** = 45)	**NLC2: 10–13** (***n*** = 45)	**NLC3: 14–17** (***n*** = 45)	**NLC4: ≥ 18** (***n*** = 55)	**P**^***a***^
Gender						0.648^b^
Male	114 (60%)	29 (64.4%)	24 (53.3%)	29 (64.4%)	32 (58.2%)	
Female	76 (40%)	16 (35.6%)	21 (46.7%)	16 (35.6%)	23 (41.8%)	
Age, years (SD)	63.7 ± 12.7	67.6 ± 11.3	63.5 ± 12.3	62.3 ± 12.3	61.8 ± 14.2	0.115^c^
Age, years						0.017*^b^
≤ 60	79 (41.6%)	10 (22.2%)	19 (42.2%)	24 (53.3%)	26 (47.3%)	
> 60	111 (58.4%)	35 (77.8%)	26 (57.8%)	21 (46.7%)	29 (52.7%)	
Chemotherapy						
Yes	125(65.8%)	34(75.6%)	31(68.9%)	30(66.7%)	30(54.5%)	0.157^**b**^
No	65(34.2%)	11(24.4%)	14(31.1%)	15(33.3%)	25(45.5%)	
Tumor location						0.014*^b^
Right side colon	76 (40%)	11 (24.4%)	20 (44.4%)	16 (35.6%)	29 (52.7%)	
Left side colon	45 (23.7%)	8 (17.8%)	10 (22.2%)	15 (33.3%)	12 (21.8%)	
Rectum	69 (36.3%)	26 (57.8%)	15 (33.3%)	14 (31.1%)	14 (25.5%)	
Pathology						0.812^d^
Adenocarcinoma	178 (93.7%)	41 (91.1%)	43 (95.6%)	43 (95.6%)	51 (92.7%)	
Mucinous adenocarcinoma	12 (6.3%)	4 (8.9%)	2 (4.4%)	2 (4.4%)	4 (7.3%)	
Differentiation grade						0.180^d^
High	6 (3.2%)	2 (4.4%)	1 (2.2%)	0 (0%)	3 (5.5%)	
Middle	146 (76.8%)	33 (73.3%)	40 (88.9%)	35 (77.8%)	38 (69.1%)	
Low	38 (20%)	10 (22.2%)	4 (8.9%)	10 (22.2%)	14 (25.5%)	
T stage						0.388^d^
pT1-2	8 (4.2%)	2 (4.4%)	1 (2.2%)	4 (8.9%)	1 (1.8%)	
pT3-4	182 (95.8%)	43 (95.6%)	44 (97.8%)	41 (91.1%)	54 (98.2%)	
N stage						< 0.001*^b^
pN0	102 (53.7%)	10 (22.2%)	19 (42.2%)	29 (64.4%)	44 (80%)	
pN1	48 (25.3%)	11 (24.4%)	16 (35.6%)	14 (31.1%)	7 (12.7%)	
pN2	40 (21.1%)	24 (53.3%)	10 (22.2%)	2 (4.4%)	4 (7.3%)	
TNM stage						< 0.001*^d^
I	8 (4.2%)	2 (4.4%)	1 (2.2%)	4 (8.9%)	1 (1.8%)	
II	94 (49.5%)	8 (17.8%)	18 (40%)	25 (55.6%)	43 (78.2%)	
III	88 (46.3%)	35 (77.8%)	26 (57.8%)	16 (35.6%)	11 (20%)	
Total lymph node, Median (IQR)	15 (12, 19)	10 (8, 12)	13 (12, 14)	16 (15, 17)	23 (20, 27)	< 0.001*^e^
< 12	31 (16.3%)	26 (57.8%)	5 (11.1%)	0 (0%)	0 (0%)	< 0.001*^d^
≥ 12	159 (83.7%)	19 (42.2%)	40 (88.9%)	45 (100%)	55 (100%)	
Lymph node ratio						< 0.001*^d^
LNR1: < 0.12	131 (68.9%)	16 (35.6%)	26 (57.8%)	39 (86.7%)	50 (90.9%)	
LNR2: 0.12—0.18	19 (10%)	3 (6.7%)	8 (17.8%)	4 (8.9%)	4 (7.3%)	
LNR3: 0.18—0.4	23 (12.1%)	9 (20%)	11 (24.4%)	2 (4.4%)	1 (1.8%)	
LNR4: > 0.4	17 (8.9%)	17 (37.8%)	0 (0%)	0 (0%)	0 (0%)	
Tumor size, cm (SD)	5.2 ± 2.1	4.9 ± 2.0	4.6 ± 1.8	5.5 ± 2.4	5.7 ± 1.8	0.026*^c^
≤ 5	113 (59.5%)	28 (62.2%)	32 (71.1%)	24 (53.3%)	29 (52.7%)	0.220^b^
> 5	77 (40.5%)	17 (37.8%)	13 (28.9%)	21 (46.7%)	26 (47.3%)	
Surgery						0.238^b^
Open surgery	146 (76.8%)	39 (86.7%)	35 (77.8%)	31 (68.9%)	41 (74.5%)	
Laparoscopic surgery	44 (23.2%)	6 (13.3%)	10 (22.2%)	14 (31.1%)	14 (25.5%)	
Follow-up (months), Mean, 95% CI	74(36, 95.8)	28 (20, 65)	61(29, 96)	80 (64, 97)	90 (71.5, 107.5)	< 0.001*^e^

*Abbreviations: NLC* Negative lymph node count, *LNR* Lymph node ratio, *SD* Standard Deviation, *IQR* Interquartile Range [25%-75%]

^a^*P* values indicate differences among NLC1, NLC2, NLC3 and NLC4

^b^Pearson's χ2 test

^c^ANOVA test

^d^Fisher’s exact test

^e^Kruskal-Wallis test

^*^P < 0.05

### Survival analysis

**Fig. 2:**
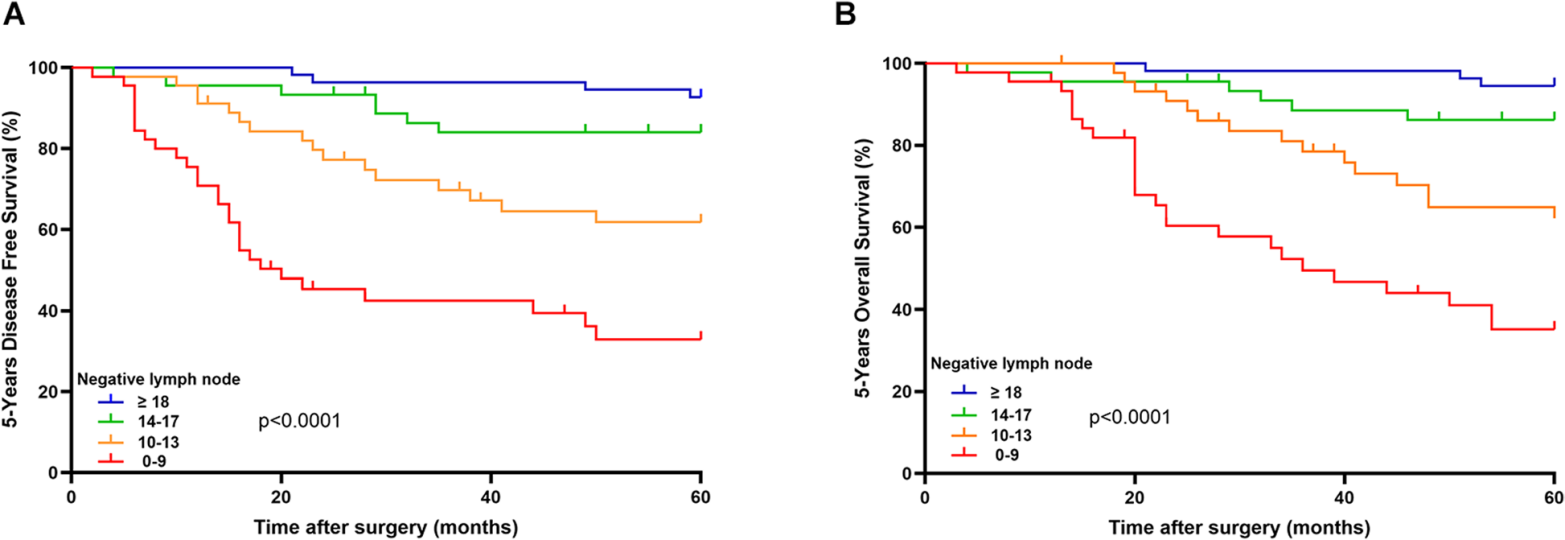
Kaplan–Meier survival curves according to the number of negative lymph node count of MSI-H/dMMR colorectal cancer patients in (**A**) 5-years disease free survival and (**B**) 5-years overall survival

The mean follow-up time was 74 months (95% CI, 36—95.8 months), and this study included 60 months of survival data for prognosis analysis (Table 1). We assessed disease free survival (DFS) and overall survival (OS) according to the number of negative lymph nodes (Fig. 2). Both five-year DFS and OS were significantly higher with an increasing number of negative lymph nodes (log-rank test *P* < 0.0001).

5-year DFS rate and 5-year OS rate were analyzed according to I-III TNM stage. In total I-III stage, increased negative lymph node counts corresponded to elevated 5-years DFS rate (37.8%, 64.4%, 84.4% and 92.7%, respectively, *p* < 0.001) and 5-years OS rate (42.2%, 66.7%, 86.7% and 94.5%, respectively, *p* < 0.001) (Table 2 and Table 3). Stratified analysis was performed based on different TNM stages, and in both stage II and stage III patients, the 5-year DFS rate and 5-year OS rate also increased with higher negative lymph node count. In stage I subgroup patients, due to the limited number of cases (8 patients) and reduced occurrence of tumor recurrence or mortality, no statistically significant differences were observed between different groups.

**Table 2:** 5-year disease free survival rates according to number of negative lymph nodes and stage (R0 resection)

**TNM Stage**	**All cases** (***n*** = 190)	**Negative lymph node count (5-year DFS rate, %)**				
		**NLC1: 0–9** (***n*** = 45)	**NLC2:10–13** (***n*** = 45)	**NLC3:14–17** (***n*** = 45)	**NLC4: ≥ 18** (***n*** = 55)	**P**^**a**^
I-III	70.1%	37.8%	64.4%	84.4%	92.7%	**< 0.001**^b^*
I	87%	50%	100%	100%	100%	0.33^c^
II	84%	62.5%	61.1%	88%	95.3%	**0.003**^b^*
III	55.7%	31.4%	65.4%	75%	81.8%	**0.002**^b^*

*Abbreviations*: *NLC* Negative lymph node count, *DFS* Disease free survival

^a^*P* values indicate differences among NLC1, NLC2, NLC3 and NLC4

^b^Pearson's χ^2^ test

^c^ Fisher’s exact test

^*^P < 0.05

**Table 3:** 5-year overall survival rates according to number of negative lymph nodes and stage (R0 resection)

**TNM Stage**	**All cases** (***n*** = 190)	**Negative lymph node count (5-year OS rate, %)**				
		**NLC1: 0–9** (***n*** = 45)	**NLC2:10–13** (***n*** = 45)	**NLC3:14–17** (***n*** = 45)	**NLC4: ≥ 18** (***n*** = 55)	**P **^**a**^
I-III	73.7%	42.2%	66.7%	86.7%	94.5%	**< 0.001**^b^*
I	87.5%	50%	100%	100%	100%	0.33^c^
II	85.3%	62.5%	66.7%	92%	95.3%	**0.004**^b^*
III	59.1%	37.1%	65.4%	75%	90.9%	**0.003**^b^*

*Abbreviations: NLC* Negative lymph node count, *OS* Overall survival

^a^*P* values indicate differences among NLC1, NLC2, NLC3 and NLC4

^b^Pearson's χ^2^ test

^c^ Fisher’s exact test

^*^P < 0.05 was considered statistically significant

**Fig. 3:**
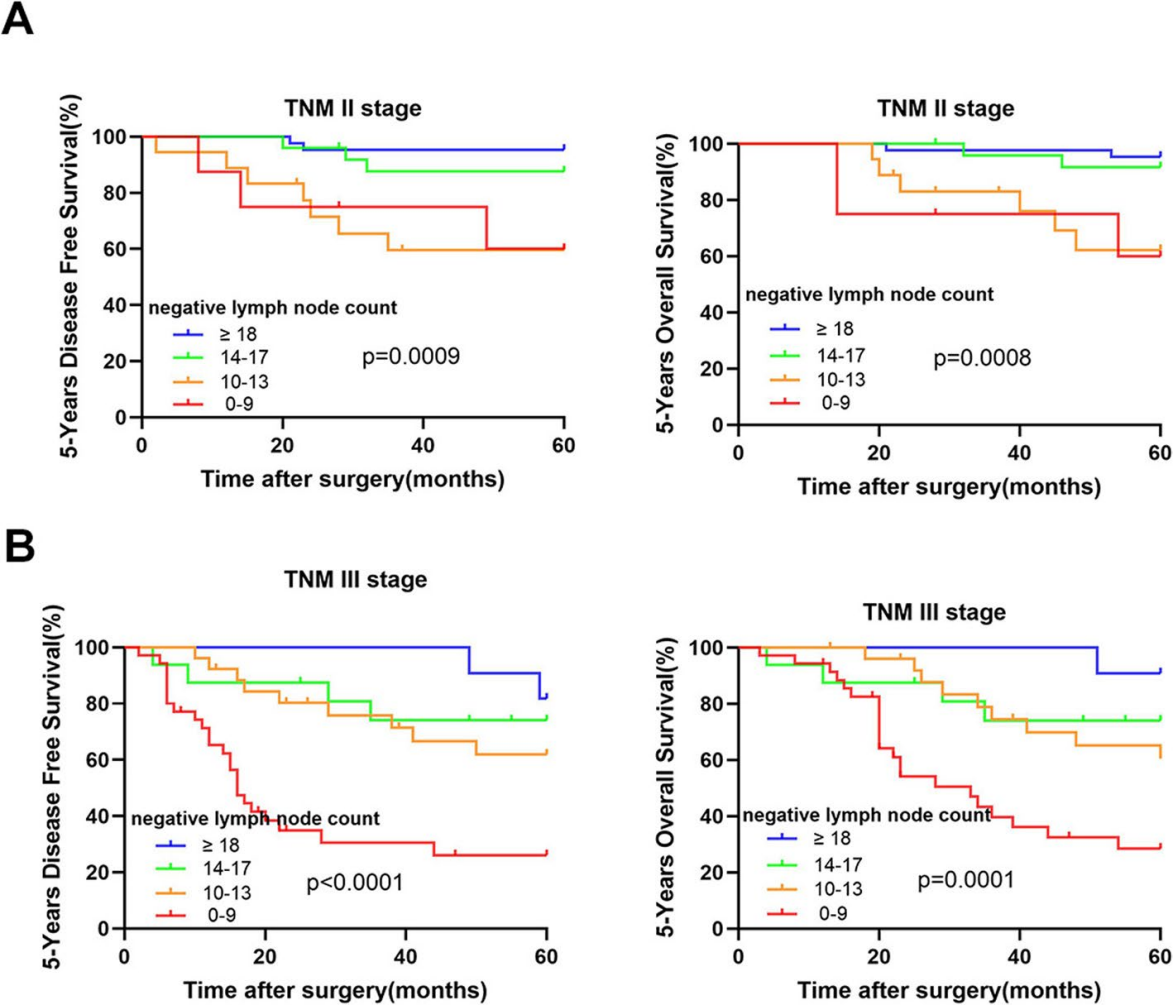
Kaplan–Meier survival curves according to the number of negative lymph node count in (**A**) stage II patients and (**B**) stage III patients

In addition, prognostic data of Kaplan–Meier curves were also analyzed in stage II and stage III patients, respectively (Fig. 3). Similarly, there was a significant difference in Kaplan–Meier curves between the different NLC subgroups (log-rank test, *p* < 0.05).

### Univariate and multivariate analyses of clinicopathologic variables in relation to DFS and OS in MSI-H/dMMR CRC patients

The clinicopathological variables, including age, gender, tumor location, pathology, differentiation grade, tumor size, chemotherapy status, T stage, N stage, LNR, total lymph node and negative lymph node count, were tested using univariate and multivariate COX regression analysis for DFS (Table 4) and OS (Table 5).

**Table 4:** Univariate and multivariate analyses of clinicopathologic variables in relation to DFS in MSI-H/dMMR CRC patients

**Variable**	**Univariate analysis of DFS**			**Multivariate analysis of DFS**	
	**HR (95% CI)**	**P**		**HR (95% CI)**	**P**
Gender					
Male	1				
Female	0.876 (0.508–1.509)	0.632		0.784(0.435—1.413)	0.418
Age, years					
≤ 60	1				
> 60	1.938 (1.083–3.470)	0.026*		1.613(0.834—3.122)	0.156
Chemotherapy					
No	1			1	
Yes	2.163 (1.140–4.103)	0.018*		1.447(0.663—3.160)	0.353
Tumor location					
Right side colon	1				
Left side colon	0.758 (0.359–1.600)	0.467		0.804(0.358—1.805)	0.596
Rectum	1.226 (0.683–2.201)	0.494		0.710(0.366—1.378)	0.312
Pathology					
Adenocarcinoma	1				
Mucinous adenocarcinoma	2.313 (0.990–5.405)	0.053		2.066(0.588—7.263)	0.258
Differentiation grade					
High	1				
Middle	0.986 (0.238–4.082)	0.985		0.641(0.128—3.214)	0.589
Low	1.288 (0.290–5.708)	0.739		0.830(0.136—5.077)	0.840
Tumor size, cm					
≤ 5	1				
> 5	0.893 (0.518–1.538)	0.682		1.048(0.571—1.924)	0.881
T stage					
pT1-2	1				
pT3-4	2.929 (0.405–21.177)	0.287		2.372(0.260—21.657)	0.444
N stage					
pN0	1				
pN1	2.578 (1.288–5.160)	0.007*		1.357(0.523—3.519)	0.530
pN2	5.650 (2.973–10.735)	< 0.001*		2.371(0.714—7.869)	0.159
Lymph node ratio					
LNR1: < 0.12	1				
LNR2: 0.12—0.18	1.884 (0.778–4.565)	0.161		1.144(0.363—3.605)	0.819
LNR3: 0.18—0.4	2.166 (0.982–4.775)	0.055		0.571(0.186—1.756)	0.328
LNR4: > 0.4	9.874 (5.053–19.297)	< 0.001*		1.033(0.230—4.642)	0.966
Total lymph node					
< 12	1				
≥ 12	0.350 (0.195–0.628)	< 0.001*		1.113(0.398—3.118)	0.838
Negative lymph node count					
NLC1: 0–9	1				
NLC2: 10–13	0.372 (0.200–0.690)	0.002*		0.386(0.130—1.145)	0.086
NLC3: 14–17	0.138 (0.060–0.319)	< 0.001*		0.169(0.042—0.681)	0.012*
NLC4: ≥ 18	0.060 (0.021–0.171)	< 0.001*		0.063(0.013—0.298)	< 0.001*

*Abbreviations: NLC* Negative lymph node count, *LNR* Lymph node ratio, *DFS* Disease free survival

^*^P < 0.05

**Table 5:** Univariate and multivariate analyses of clinicopathologic variables in relation to OS in MSI-H/dMMR CRC patients

Variable	Univariate analysis of OS		Multivariate analysis of OS	
	**HR (95% CI)**	**P**	**HR (95% CI)**	**P**
Gender				
Male	1			
Female	0.879 (0.497–1.556)	0.659	0.768(0.412—1.431)	0.406
Age, years				
≤ 60	1			
> 60	2.298 (1.221–4.324)	0.01*	2.060(0.990—4.287)	0.053
Chemotherapy				
No	1		1	
Yes	2.109 (1.080–4.120)	0.029*	1.426(0.624—3.258)	0.400
Tumor location				
Right side colon	1			
Left side colon	0.875 (0.407–1.882)	0.733	0.763(0.334—1.739)	0.519
Rectum	1.248 (0.671–2.322)	0.484	0.554(0.268—1.144)	0.111
Pathology				
Adenocarcinoma	1			
Mucinous adenocarcinoma	1.910 (0.758–4.813)	0.170	2.033(0.554—7.459)	0.285
Differentiation grade				
High	1			
Middle	0.894 (0.215–3.713)	0.877	0.570(0.110—2.967)	0.504
Low	1.140 (0.255–5.097)	0.864	0.675(0.107—4.259)	0.676
Tumor size, cm				
≤ 5	1			
> 5	0.81 (0.455–1.443)	0.475	1.091(0.575—2.072)	0.790
T stage				
pT1-2	1			
pT3-4	2.639 (0.364–19.119)	0.337	2.011(0.208—19.428)	0.546
N stage				
pN0	1			
pN1	3.048 (1.486–6.252)	0.002*	1.905(0.734—4.943)	0.185
pN2	5.323 (2.681–10.568)	< 0.001*	2.895(0.803—10.431)	0.104
Lymph node ratio				
LNR1: < 0.12	1			
LNR2: 0.12—0.18	1.666 (0.637–4.354)	0.298	0.632(0.201—1.987)	0.432
LNR3: 0.18—0.4	2.410 (1.085–5.352)	0.031*	0.409(0.124—1.352)	0.143
LNR4: > 0.4	7.161 (3.564–14.388)	< 0.001*	0.411(0.087—1.934)	0.260
Total lymph node				
< 12	1			
≥ 12	0.369 (0.198–0.686)	0.002*	2.335(0.797—6.838)	0.122
Negative lymph node count				
NLC1: 0–9	1			
NLC2: 10–13	0.401 (0.212–0.759)	0.005*	0.217(0.071—0.669)	0.008*
NLC3: 14–17	0.136 (0.056–0.331)	< 0.001*	0.076(0.018—0.330)	0.001*
NLC4: ≥ 18	0.050 (0.015–0.166)	< 0.001*	0.025(0.005—0.137)	< 0.001*

*Abbreviations*: *NLC* Negative lymph node count, *LNR* Lymph node ratio, *OS:* Overall survival

^*^P < 0.05

Univariate analysis found that age, chemotherapy status, N stage, LNR, total lymph node and NLC were all confirmed to be prognostic predictive factor for tumor recurrence and associated with DFS (Table 4). In multivariate analysis, only NLC (NLC2, HR = 0.386, *P* = 0.086; NLC3, HR: 0.169, *P* = 0.012; NLC4, HR: 0.063, *P* < 0.001) is the independent prognostic factor for tumor recurrence. However, age, chemotherapy status, N stage, LNR and total lymph node were not found to be related to DFS in multivariate analysis.

For OS, in univariate analysis, age, chemotherapy status, N stage, LNR, total lymph node and NLC were all considerably correlated with OS (Table 5). In multivariate analysis, only NLC (NLC2, HR: 0.217, *P* = 0.008; NLC3, HR: 0.076, *P* = 0.001; NLC4, HR: 0.025, *P* < 0.001) is the protective factor for 5-year death.

### ROC analysis and comparison for different predictive indicators

**Fig. 4:**
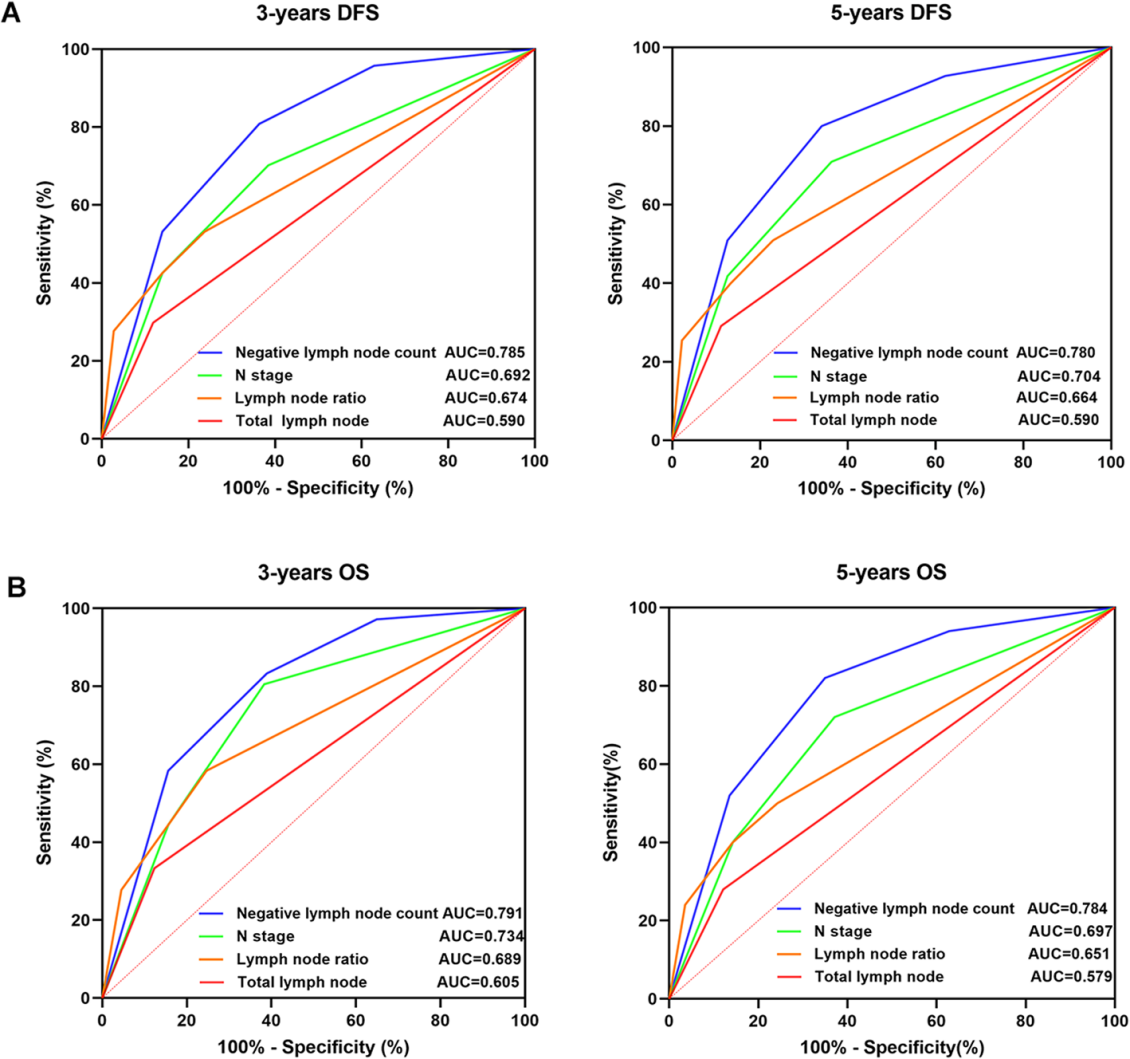
Comparison of ROC curves for NLC, N stage, LNR and total lymph node. The AUC, which defined as the geometric area to the lower right of the ROC, is used to quantify the predictive efficacy in DFS and OS, with values between 0.7 and 0.9 indicating moderate predictive efficacy. **A** ROC curve for 3-years and 5-years DFS; (**B**) ROC curve for 3-years and 5-years OS

In order to compare the prognostic predictive efficacy of NLC, N stage, LNR and total lymph node for MSI-H/dMMR patients, we employed the ROC (Receiver Operating Characteristic Curve) method to evaluate the sensitivity and specificity of different predictive indicators. The AUC (Area Under Curve), which defined as the geometric area to the lower right of the ROC, is used to quantify the predictive efficacy in DFS and OS, with the values between 0.7 and 0.9 indicating moderate predictive efficacy. Figure 4 showed the comparison between NLC and LNR, total lymph node, N stage in 3-years and 5-years survival predicting. The AUC of NLC (3-years DFS:0.785, 5-years DFS:0.780, 3-years OS:0.791, 5-years OS:0.784) were significantly higher than N stage (3-years DFS:0.692, 5-years DFS:0. 704, 3-years OS:0.734, 5-years OS:0.697), LNR (3-years DFS:0.674, 5-years DFS:0.664, 3-years OS:0.689, 5-years OS:0.651) and total lymph node (3-years DFS:0.590, 5-years DFS:0. 590, 3-years OS:0.605, 5-years OS:0.579).

### C-index analysis and change in the C-index for different predictive indicators

Additionally, we employed C-index (Concordance index) analysis to conduct pairwise comparisons of different predictive indicators for DFS and OS, aiming to evaluate whether NLC exhibited superior predictive efficacy. C-index in the range of 0.7 to 0.9 is indicative of moderate predictive efficacy. The C-index of NLC (DFS:0.769; OS:0.773) in survival predicting were significantly higher than TLN (DFS:0.583; OS:0.579), LNR (DFS:0.666; OS:0.655) and N stage (DFS:0.693; OS:0.691) (Table 6). Statistically significant differences were observed in the C-index change when comparing NLC with other predictive indicators.

**Table 6:** Comparison between NLC and TLN, LNR, N stage in Concordance index (C-index)^a^

	**C-index**	**95% CI**	**P value**
**Prediction of DFS**			
Negative lymph node count (VS.TLN)	0.769	0.715—0.823	
TLN	0.583	0.527- 0.640	
C-index change	0.186	0.122—0.248	< 0.001*
Negative lymph node count (VS.LNR)	0.769	0.715—0.823	
LNR	0.666	0.598—0.733	
C-index change	0.103	0.041—0.177	0.003*
Negative lymph node count (VS. N stage)	0.769	0.715—0.823	
N stage	0.693	0.627—0.759	
C-index change	0.076	0.011—0.143	0.024*
**Prediction of OS**			
Negative lymph node count (VS.TLN)	0.773	0.715—0.832	
TLN	0.579	0.519—0.639	
C-index change	0.195	0.129—0.261	< 0.001*
Negative lymph node count (VS.LNR)	0.773	0.715—0.832	
LNR	0.655	0.583—0.727	
C-index change	0.118	0.051—0.195	0.001*
Negative lymph node count (VS. N stage)	0.773	0.715—0.832	
N stage	0.691	0.622—0.760	
C-index change	0.082	0.013—0.153	0.022*

*Abbreviations: NLC* Negative lymph node count, *LNR* Lymph node ratio, *TLN* Total lymph node, *C-index* concordance index, *DFS* Disease free survival, *OS* Overall survival

^a^C-index is employed to assess the predictive capability of prognostic indicators, primarily utilized in the survival analysis to measure the discrimination between COX model predicted values and actual outcomes. The C-index represents the proportion of patients whose predicted outcomes match the actual outcomes among the entire patient population. The C-index change enables comparison of prediction accuracy between two prediction models. The C-index of 0.5–0.7 represented low predictive efficacy, 0.7–0.9 indicated moderate predictive efficacy, and > 0.9 represented high predictive efficacy

^*^P < 0.05

## Discussion

In the presence of mismatch repair genes deficiency (MLH1, MSH2, MSH6, PMS2), replication errors occur and accumulate in DNA microsatellites (MS), resulting in alterations in the base sequence of MS, referred to as microsatellite instability (MSI) [2]. MSI-H/dMMR also lead to an elevated tumor mutation burden (TMB), consequently increase the risk of tumorigenesis, which is one of the important oncogenic pathways in colorectal cancer (CRC) [21]. MSI-H/dMMR patients account for 5% to 15% of the total CRC cases, who have distinct and unique characteristics as follows [2]: (1) younger age; (2) higher prevalence in the right colon; (3) elevated proportion of mucinous adenocarcinoma; (4) localized tumor growth with larger tumor size; (5) higher incidence of total lymph node harvest and lower incidence of lymph node metastasis, with a relatively higher rate of stage II cases.

MSI-H cancer is also referred to as "hot tumor". Deficiency of mismatch repair genes leads to higher tumor mutation burden and the expression of "neoantigens" on the surface of tumor cells. These neoantigens enhance the recognition of the tumor by the immune system, thus provoking a strong immune response and lymphocyte infiltration into the tumor microenvironment. Due to the strong immunogenicity, MSI-H/dMMR patients are less likely to develop lymph node or distant metastasis, with strong lymph node reaction and higher total lymph nodes harvested [9–12]. Studies have shown that higher lymph node harvest in MSI-H/dMMR resection specimens indicates stronger immune response and more favorable prognosis [9].

Due to the lower likelihood of lymph node metastasis in MSI-H/dMMR patients and the higher prevalence of stage II cases, the predictive value of traditional N stage and LNR is limited. For instance, many MSI-H/dMMR patients with locally advanced tumors still do not develop lymph node metastases, thus, the N stage or LNR (remain 0 in I-II stage) fail to reflects the patients' immune status and survival prognosis. In MSI-H/dMMR patients with high immunogenicity, there is a close relationship between lymph node count and prognosis. The calculation of negative lymph nodes count (NLC), which takes into account both the total lymph node and the metastatic lymph node, serves as a strong prognostic indicator.

We examined the prognostic significance of the negative lymph node count in 190 MSI-H/dMMR CRC patients who received R0 curative resection. We observed higher NLC were associated with better survival prognosis in MSI-H/dMMR patients, and it exhibited a stronger predictive power for 5-years DFS and OS compared to N stage, LNR and total lymph node harvest. In addition, NLC is the only independent prognostic factor for tumor recurrence and death after adjusting the various clinical and pathologic features in multivariate analysis. Furthermore, we observed from Table 1 that patients with greater negative lymph node count exhibited younger age, higher prevalence of right-sided tumor localization, and larger tumor sizes. These characteristics were more consistent with the clinical features of MSI-H patients, and may be associated with the oncogenic pathway of dMMR and activation of host lymphocytic reaction to tumor.

For the pathological results of MSI-H patients after surgery, more attention should be paid to the NLC in addition to positive lymph nodes. A lower NLC correlates with poorer prognosis. Particularly for patients with NLC ranging from 0 to 9, they represent a high-risk group for tumor recurrence and should undergo close postoperative follow-up examinations.

The mechanism underlying the survival advantage associated with the negative lymph node count remains uncertain. The number of lymph nodes may be an indicator of host immune response to tumor cells [18, 19]. The benefit associated with a higher negative lymph node count may reflect the host lymphocytic reaction to tumor. While LNR also served as a valuable prognostic indicator, its predictive efficacy was inferior to NLC, primarily because it cannot predict the prognosis of patients without lymph node metastasis, since the LNR value remained at 0 in stage I—II patients.

It is worth noting that we have not identified predictive value of NLC in MSS patients. MSS colorectal carcinomas differ from MSI cancer in terms of underlying genetic pathway and clinical-pathological features. MSS patients represent the anti-tumor immune inactive subgroup, characterized by proficiency of mismatch repair genes (pMMR), low tumor mutational burden and low expression of "neoantigens", resulting in weaker antitumor immune response, also referred to as the “cold tumors” [22]. The correlation between the anti-tumor immune response and lymph nodes is less pronounced in MSS/pMMR patients compared to MSI-H/dMMR patients [23–25].

There are limitations in this study. First, this is a retrospective study with small sample size. The results were preliminary. Prospective validations in large cohorts are required. Second, this study aims to investigate the correlation between NLC and the prognosis in MSI patients, clarifying whether it is a stronger prognostic factor, rather than constructing a predictive model for the prognosis. Numerous factors influence a patient's prognosis, and the lymph node is merely one of the important prognostic factors. The C-index for NLC in terms of DFS and OS were 0.769 and 0.775, respectively, which indicated a moderate predictive capability.

## Conclusion

Negative lymph node is an important independent prognostic factor for MSI-H CRC patients. Reduced NLC is associated with tumor recurrence and poor survival, which is a stronger prognostic factor than N stage, TLN and LNR. Closer postoperative follow-up and more active clinical interventions should be considered for MSI-H patients with low NLC. Our data imply a possible role of host immune response as an independent prognostic factor in MSI-H/dMMR CRC patients. Future studies are needed to validate these findings and elucidate the underlying mechanisms through which the lymphocytic response impacts the clinical outcome in MSI-H/dMMR CRC.

## Data Availability

The data presented in this study are available on reasonable request from the corresponding author. The data are not publicly available due to privacy.

## Abbreviation

NLC: Negative lymph nodes count
LNR: Lymph node ratio
OS: Overall survival
DFS: Disease free survival
ROC: Receiver operating characteristic curve
AUC: Area under curve
